# Age‐ and disease‐related autophagy impairment in Huntington disease: New insights from direct neuronal reprogramming

**DOI:** 10.1111/acel.14285

**Published:** 2024-07-23

**Authors:** Chuyang Luo, Junsheng Yang

**Affiliations:** ^1^ Collaborative Innovation Center of Yangtze River Delta Region Green Pharmaceuticals College of Pharmaceutical Sciences, Zhejiang University of Technology Hangzhou China

**Keywords:** aging, autophagy, direct neuronal reprogramming, Huntington disease

## Abstract

Autophagy impairment in Huntington disease (HD) has been reported for almost two decades. However, the molecular mechanisms underlying this phenomenon are still unclear. This is partially because it is challenging to model the impact of the disease‐causing mutation, aging, as well as the selective vulnerability of neurons in a single model. Recently developed direct neuronal reprogramming that allows researchers to induce neurons‐of‐interest retaining biological aging information made it possible to establish HD cellular models to study more relevant age‐ and disease‐related molecular changes in neurons. We here summarized the findings from a few latest studies utilizing directly reprogrammed HD neurons and discussed the new insights they brought to the understanding of the age‐ and disease‐related autophagy impairment in HD.

## Graphical abstract

It is challenging to model the impact of aging in age‐related disease like Huntington disease (HD). Recently developed direct neuronal reprogramming allows researchers to induce neurons‐of‐interest retaining biological aging information. We here summarized the findings from a few latest studies utilizing directly reprogrammed HD neurons and discussed the new insights they brought to the understanding of the age‐ and disease‐related autophagy impairment in HD.

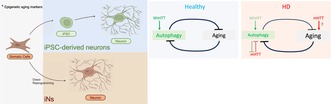

**Abbreviations**
CaN: calcineurin
CDM: *Ctip2, Dlx1, Dlx2, Myt1l*
CN: cortical neuron
DAR: differently accessible region
GLB: glibenclamide
HD: Huntington Disease
*HTT*
: huntingtin gene
iPSCs: induced pluripotent stem cells
mHTT: mutant huntingtin
MSN: medium spiny neuron
mTOR: mechanistic target of rapamycin
REST1: RE1‐silencing transcription factor
ROS: reactive oxygen species
shRNA: short hairpin RNAs
STAT3: signal transducers and activators of transcription 3
TF: transcriptions factor
TFEB: Transcription Factor EB
WtHTT: wild type huntingtin

Autophagy, a lysosome‐mediated process that degrades a variety of materials ranging from macromolecules to organelles, plays crucial roles in maintaining cellular homeostasis and is linked to a plethora of human diseases (Klionsky et al., 2021; Mizushima & Levine, 2020). Autophagy has also been reported to be compromised in neurodegenerative diseases including Huntington disease (HD), an autosomal dominant monogenic neurodegenerative disease caused by the expansion of CAG repeats in the first exon of huntingtin gene (*HTT*) that produce aggregation‐prone mutant huntingtin protein (mHTT) (Giovedì et al., 2020; Menzies et al., 2015). Although it was believed that boosting autophagy should be beneficial for HD, attempts to reduce mHTT and alleviate HD pathology by improving autophagy were not all successful (Djajadikerta et al., 2020; Yang & Zhang, 2023), possibly due to a few reasons. First, autophagy is a multi‐step process requiring the coordination of autophagosome and lysosome that is influenced by complex environmental cues including nutrient availability and stresses via key regulators such as the mechanistic target of rapamycin (mTOR) and transcription factor EB (TFEB). mHTT has been reported to interfere the autophagy process at multiple levels including the transcription of key autophagy genes, cargo recognition and lysosome trafficking (Yang & Zhang, 2023). The role of wild‐type huntingtin (WtHTT) as a scaffold protein in selective autophagy added yet another layer of complexity to the autophagy problem in HD (Rui et al., 2015). It is thus necessary to understand more accurately the molecular mechanisms underlying the autophagy defects in HD before one can target autophagy more precisely to possibly treat HD. Second, many of the HD cell models used in autophagy‐related studies were mitotic cells overexpressing mHTT, either full length or a fragment, with a long expanded polyQ (e.g., over 100Q), which is not commonly seen in HD patients. It is also worth clarifying that models overexpressing merely polyQ peptide should be considered as general models for the common features of polyglutamine diseases rather than HD models (Bonsor et al., 2024; Shao & Diamond, 2007), These models thus may only very limitedly reflect the autophagy impairment in affected neurons in HD (Rangel‐Barajas & Rebec, 2018). Third, the specific strategy in activating autophagy could be pivotal (e.g., an overall increase of autophagy by targeting key transcription factors vs. a specific boost of relevant subtypes of autophagy such as aggrephagy, mitophagy, and so on; an increase in autophagosome formation or an up‐regulation of lysosome biogenesis and activity), as well as the timing of the autophagy manipulation can largely affect the outcome (Brattås et al., 2021). Taken together, although earlier studies demonstrated autophagy a promising process to target in HD, more relevant HD models are needed to uncover the precise age‐related autophagic changes in the affected brain regions.

A few hallmark features of HD make it difficult to recapitulate its multifaceted manifestation in cellular or animal models. First, even the disease‐causing mutant have been discovered for three decades (MacDonald et al., 1993), the understanding of the molecular pathogenesis of HD is still developing (Tabrizi et al., 2020). In addition to mHTT protein, increased somatic instability and transcription silencing (Malik et al., 2021) induced by CAG repeat expansion at the DNA level and deregulated splicing and silencing by mHTT RNA may also contribute to the disease development (Banez‐Coronel et al., 2012; Qawasmi et al., 2019; Schilling et al., 2019). Second, the molecular mechanisms underlying the selective vulnerability of striatal medium spiny neurons (MSNs) to degeneration is also unclear (Morigaki & Goto, 2017). Third, the disease onset of HD depends on both the number of CAG repeats and the age of the patient. Although great progress has been made on the age‐related changes of cellular functions, especially protein homeostasis in health and disease (Hipp et al., 2019; Hipp & Hartl, 2024; Llewellyn et al., 2024), the complex interplay among the loss‐of‐function of the WtHtt, the gain‐of‐function of mHTT, and autophagy is still not fully understood. Taken together, models that can more comprehensively reflex the age‐ and disease‐related biological changes in HD patients' brains, including autophagy impairment, are still in great need.

Induced pluripotent stem cells (iPSCs) are powerful tools in modeling diseases due to their reprogrammable nature into diverse cell types including neurons. However, iPSCs have a critical limitation when modeling age‐dependent diseases such as HD as they exhibit an embryonic state that fail to retain age‐related characteristics (Lapasset et al., 2011; Patterson et al., 2012). Alternatively, multiple groups separately reported methods to directly reprogram human adult fibroblasts to neurons by forced expression of certain sets of transcriptions factors (TFs) or miRNA/TF combinations in 2011 (Ambasudhan et al., 2011; Caiazzo et al., 2011; Pang et al., 2011; Pfisterer et al., 2011; Yoo et al., 2011). Following studies not only continued improving the efficiency of fibroblast‐to‐functional‐neuron conversion (see a recent review; Bocchi et al. 2022), but also successfully generated specific subtypes of neurons such as MSNs by co‐expressing miR‐9/9*‐124 and transcription factors *Ctip2*, *Dlx1*, *Dlx2*, *Myt1l* (CDM) (Drouin‐Ouellet et al., 2017; Victor et al., 2014). More importantly, directly reprogrammed neurons appeared to be capable of by‐passing the pluripotent state and retaining age‐related biological changes such as increased ROS, accumulated DNA damage, shortened telomeres, increased DNA‐methylation‐based “epigenetic age,” age‐dependent transcriptomic signatures and microRNA profiles from the donors' skin fibroblasts (Huh et al., 2016; Mertens et al., 2015). Although the roles of some of these biological changes in aging are still highly debatable (e.g., an increase in ROS did not necessarily induce aging; Lee et al., 2010; van Soest et al., 2024), the capability of directly reprogrammed neurons to remain such changes makes them particularly valuable tools for the modeling of age‐related diseases like HD. Unlike iPSC‐derived HD neurons that require additional conditions to induce key HD biomarkers such as mHTT aggregation, directly reprogrammed HD neurons spontaneously displayed mHTT aggregation along with other disease‐associated phenotypes including DNA damage, mitochondrial dysfunction, neuronal maturate impairment and spontaneous degradation (Drouin‐Ouellet et al., 2017; Liu et al., 2014; Victor et al., 2018) (and also Monk and Connor (2021) for a comprehensive review). Furthermore, a few latest studies utilizing this approach have confirmed the autophagy impairment in HD and brought novel insights to the underlying mechanisms.

In a study published in 2022 that compared the transcriptomes of miR‐9/9*‐124‐CDM‐based MSNs directly reprogrammed from skin fibroblasts of healthy young and old, as well as young pre‐HD and old symptomatic HD individuals with similar CAG repeat numbers (with 6 independent samples in each group), Oh et al. (2022) found an age‐related decline of autophagy‐related genes specifically in the old‐HD‐MSNs but not in the old‐healthy‐MSNs. In line with this, old‐HD‐MSNs manifested impaired autophagy activity, indicated by decreased autophagosomes, autolysosomes and accumulated p62, as well as increased apoptotic markers when compared with young‐pre‐HD‐ or healthy‐MSNs. Moreover, autophagy inhibition led to increased mHTT aggregation and cell death in young‐pre‐HD‐MSNs while boosting autophagy by G2‐115, a glibenclamide (GLB) analog, alleviated old HD‐MSNs degeneration. By further comparing differently accessible regions (DARs) in young‐pre‐HD‐MSNs and old‐HD‐MSNs' chromatins, the authors identified miR29B1, a precursor of miR29B1‐3p whose targets include autophagy‐related genes, is up‐regulated in old HD‐MSNs over young‐pre‐HD‐MSNs much more dramatically than old‐healthy‐MSNs over young‐healthy‐MSNs. Consistent with this, anti‐sense inhibition of miR29B1‐3p was sufficient to restore autophagy activity, reduce mHTT aggregation and apoptosis in old‐HD‐MSNs, while overexpressing miR29B1‐3p in young‐pre‐HD‐MSNs had the opposite effects. miR29B1‐3p appeared to reduce autophagy via directly targeting the 3'UTR of *STAT3*, which encodes signal transducers and activators of transcription 3 (STAT3), and decreasing its expression.

In another study just published by the same group (Lee et al., 2024), Lee et al. applied the same direct reprogramming method to generate MSNs from fibroblasts collected at two time points (around 50 and 70 years of age) from three healthy individuals. When longitudinally comparing the transcriptomes of the MSNs and their source fibroblasts, the authors revealed an MSN‐specific age‐related increase in the expression of *RCAN1*, an inhibitory regulator of the calcium‐ and calmodulin‐dependent phosphatase calcineurin (CaN). The RCAN1 protein levels also increased in old‐MSNs. Interestingly, in postmortem human striatums, the old group manifested higher protein but not transcript levels of RCAN1. In a separate screen on HD‐MSNs, RCAN1 also appeared as a gene whose knock‐down can reduce neuron degeneration and mHTT aggregation. The authors further identified transcription factor EB (TFEB), a well‐recognized master regulator of autophagy and lysosomal biogenesis (Napolitano & Ballabio, 2016), to be a critical downstream factor of the RCAN1‐CaN axis to mediate mHTT clearance and HD‐MSN survival.

Beside the two studies discussed above, autophagy impairment was also reported in HD‐iNs directly reprogrammed from a combined expression of Ascl1, Brn2, and two short hairpin RNAs (shRNA) inhibiting the RE1‐silencing transcription factor (REST1) in fibroblasts (Pircs et al., 2022). Interestingly, when comparing the transcriptomes of healthy‐iNs and HD‐iNs, the authors found the differentially expressed genes were not associated with any specific gene programs. On the contrary, proteome comparison revealed the down‐regulation of proteins enriched in autophagy‐related processes (e.g., the CAMKK‐AMPK pathway) in HD‐iNs. Assessment of autophagy activity by multiple microscopy‐based assays together with autophagy inducer and blocker in HD‐iNs uncovered a neurite‐specific accumulation of autolysosomes, suggestive of impaired autophagic degradation. Further experiments for the underlying mechanistic of autophagy defect in HD‐iNs ruled out incomplete HTT transcripts or mHTT aggregates as causal reagents. Transcriptional silencing of HTT, on the other hand, resulted an overall increase of LC3‐ or p62‐positive puncta but a neurite‐specific decrease of Lamp1‐positive puncta in healthy‐iNs, suggesting a regulatory role played by WtHTT in healthy neurons. Silencing of both copies of HTT (wild type and mutant) in HD‐iNs led to an increase of LC3‐positive puncta and a decrease of Lamp1‐positive puncta in both cell body and neurites. Morphological studies revealed that HD‐iNs displayed a reduction of neurite number and size, which can be further reduced upon amino acid starvation—a common method to induce autophagy.

In summary, the three studies discussed above successfully showcased the possibility of revealing age‐ and disease‐related molecular changes in HD neurons by direct neuronal reprogramming. The common discovery of defected autophagy functions as well as the sensitivity to autophagy manipulation in HD neurons assured the importance of autophagy in HD pathology. However, the different autophagy defects found in these studies also confirmed the complex interaction among aging, autophagy impairment, and HD disease onset (also see Figure 1a for an illustration). On the one hand, while the alteration of RCAN1‐CaN‐TFEB axis discovered from longitudinal comparison of healthy‐MSNs reflected a more general age‐related autophagy decline that can contribute to HD disease onset, the changes in the miR29B1‐3p‐STAT3 axis observed in symptomatic old‐HD‐MSNs might be a consequence rather than a cause of HD pathology. On the other hand, the proteomic but not transcriptomic compromise in autophagy in HD‐iNs that was in contrast to HD‐MSNs may have added yet another difference between MSN and other neurons, therefore may provide new mechanistic insights for the selective vulnerability of MSNs in HD. An earlier study by the Yoo group reported that cortical neurons (CNs) directly reprogrammed from HD fibroblasts manifested more mHTT aggregates but less DNA damage and cell death compared with HD‐MSNs (Victor et al., 2018). These HD‐CNs could serve as great controls for HD‐MSNs to uncover neuron subtype‐specific autophagy defects in HD. Additionally, now that comparing HD cells with similar length of CAG repeats (mostly 40–50) has successfully revealed age‐ and disease‐related autophagy impairment, it will also be interesting to explore whether a further expanded CAG repeat will cause similar autophagy dysfunctions, given that the length of CAG repeats is inversely correlated with the age‐of‐onset in HD (Andrew et al., 1993).

**FIGURE 1:**
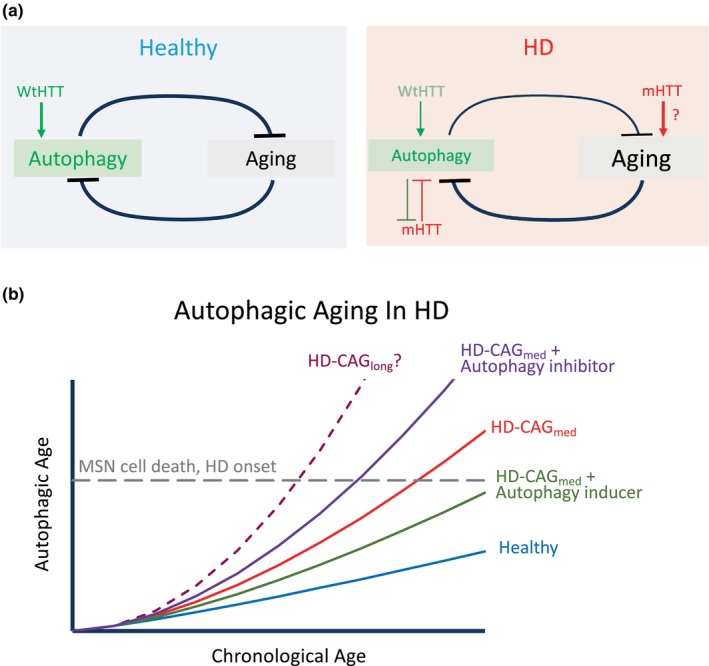
Autophagy and aging in HD. (a) The mutual antagonizing balance between autophagy and aging is broken in HD. (b) Autophagic aging is accelerated in HD and presumably correlated with both chronological age and the length of CAG repeats. Manipulating autophagy by inducers or inhibitors may decelerate or accelerate autophagic aging and interfere with MSN degeneration and HD disease onset. HD‐CAG_med_, HD patients with medium length of CAG repeats; HD‐CAG_long_, HD patients with long length of CAG repeats.

Accumulating evidence suggests that declined autophagy is not only a hallmark but also a causal factor of aging, and up‐regulating autophagy is conceivably anti‐aging (Aman et al., 2021). In HD, the scenario seems to be a vicious cycle (Figure 1a). First, the mutation in HTT will simultaneously lower the dose of wild‐type HTT (WtHTT), which facilitates autophagy (Rui et al., 2015) as well as produce mHTT, which could directly interfere with autophagy via diverse mechanisms such as lowering the activity of key transcription factors (Yang et al., 2023), reducing the initiation of autophagy (Ashkenazi et al., 2017), inhibiting the autophagosome‐lysosome fusion (Franco‐Iborra et al., 2021; Hosp et al., 2017; Pircs et al., 2018), and impairing mitophagy (Franco‐Iborra et al., 2021). Second, compromised autophagy will reduce cells' competence in clearing mHTT. Third, declined autophagy may accelerate the overall diminishing of the intracellular homeostasis during aging, which in term may further hamper autophagy and eventually led to the cell death of specific neurons.

HD patients manifest higher biological age, indicated by biomarkers such as shortened telomere in leukocytes and increased DNA methylation/epigenetic age in brains (Machiela 2020 review). The recent application of direct neuronal reprogramming in modeling HD discussed here provided valuable novel insights in the age‐related autophagy impairment, or “autophagic aging,” in MSNs and other neurons in HD (Figure 1b). Although we should still keep in mind that the aging process of the dividing fibroblasts could be quite different from the post‐mitotic neurons (Aging Biomarker et al., 2023; Moqri et al., 2023) thus the biological age signatures retained in HD patients' dermal fibroblasts may not completely illustrate the aging events happening in their brains, the reprogrammed HD‐MSNs and iNs can still serve as highly relevant tools to study not only the molecular mechanisms but also the pharmacological outcomes of autophagy manipulation in HD.

## AUTHOR CONTRIBUTIONS

CL and JY discussed the idea of this manuscript; CL prepared the initial draft; JY finalized the draft and made the figure.

## CONFLICT OF INTEREST STATEMENT

The authors declare no competing interests.

## Data Availability

Data sharing is not applicable to this article as no new data were created or analyzed in this study.
